# Priapism secondary to tamsulosin: A case report

**DOI:** 10.1016/j.ijscr.2020.06.022

**Published:** 2020-06-12

**Authors:** Johannes Cansius Prihadi, Christopher Kusumajaya

**Affiliations:** Department of Surgery, Division Urology, School of Medicine and Health Sciences, Atmajaya Catholic University, Jl. Pluit Raya No. 2, Penjaringan, Utara, DKI Jakarta 14440, Indonesia

**Keywords:** Complication, Corpora cavernosa, Detumescence, Erection, Priapism, Tamsulosin-induced-priapism

## Abstract

**Background:**

Tamsulosin is the most potent adrenergic alpha-1 antagonist used for treatment of benign prostatic hyperplasia (BPH). Priapism has been reported as a rare side effect through direct inhibition of the sympathetic input necessary for detumesence.

**Presentation of case:**

We describe an otherwise healthy man with recurrent and then persistent unresolved priapism after the use of tamsulosin and concomitant use of ace inhibitor and beta blocker for hypertension. We then performed aspiration and intracavernosal irrigation of saline and vasoconstrictive agent.

**Discussion:**

Health-care professionals should inform all patients taking such medications about this rare but possible serious adverse effect. Tamsulosin is a useful medication for the management of Lower Urinary Tract Symptoms (LUTS) related to BPH and medical expulsion of distal ureteric calculi. However, its use may be associated on rare occasions with priapism, hence Health-care professionals should be aware in order to advice all patients taking such medications about this rare but serious adverse effect and to seek help as soon as possible.

**Conclusion:**

With caution against the use of tamsulosin in hypertension treated patient, the possibility of the adverse effect can be more noticed and encourage practitioners to look for other alternatives that are safer and better for dealing with LUTS in the future and develop better treatment strategies.

## Highlights

• Priapism is a rare pathologic condition representing penile erection disorder that persist beyond 4 h or is unrelated to sexual stimulation.
• Etiology remains unknown in more than 50% of cases. In induced priapism, it caused by adverse event of drug ingestion or intracavernous injection.
• Induced priapism is rare, lack of information to patients leading to delayed medical care with its complicated consequences.
• Tamsulosin is the only alpha adrenergic blocker showing that priapism might develop as a result of the secondary effect.
• With the increasing use of Tamsulosine, it would not be surprising to see more of tamsulosin-induced-priapism cases in the future.

## 1 Introduction

Priapism is a pathologic condition representing a true disorder of penile erection that persists beyond 4 h or is unrelated to sexual interest or stimulation [1]. Priapism is a rare case, the incidence is low (1.5 cases per 100,000 person/years) [2]. Based on the episode of history, blood gas analysis, and color Doppler ultrasonography of the corpus cavernosum [3], there are three subtypes of priapisms: veno-occlusive (ischemic, low flow), intermittent (stuttering), and arterial (non-ischemic, high flow) [1,2].

The etiology of priapism remains unknown in more than 50% of cases. However, in induced priapism, the cause is known to be an adverse event or side-effect of drug ingestion or intracavernosal injection [1]. Induced priapism is rare, possibly because of this infrequency, patients inadequately informed about the possibility of the event, leading to delayed medical care with its complicated consequences. Other case reports regarding alpha-blocker-induced priapism were given in Table 1. Here, we describe a rare case of a man with prolonged persistent unresolved priapism after the use of tamsulosin who required distal aspiration and irrigation procedure to treat his ischemic priapism.

**Table 1:** Summary of all the cases of priapism associated with tamsulosin.

	Priapism cases	Follow up
Consentino et al. [1]	A 67-year-old man, episodes of priapism started after about 3–4 weeks after starting treatment for his LUTS with tamsulosin 0.4 mg No ingestion of other drugs, ortoxic substances and reported no intracavernous injection of medications or previous pelvic or abdominal trauma.	Duration of erection: 6 h. An intracavernosal injection of vasoconstrictor was performed and ultimately irrigation of the corpora cavernosa with saline solution resolved the emergency producing detumescence. Tamsulosin stopped and no further episodes of priapism.
Spagnul et al. [4]	32 years old, priapism following dosage of 0.4 mg for LUTS No concomitant diseases or use of medications. No trauma reported. No previous episodes of priapism	Duration of erection: 40 h. Priapism reverted by aspiration of the corpora and intracavernosal injection of 1:1000 adrenaline solution. Ten days later, rigid erections
Pahhuja et al. [5]	56 years old. Erection following a 2-week regimen of 0.4 mg of tamsulosin for LUTS. No illegal drugs or alcohol associated	Duration of erection: 28 h. Treated unsuccessfully with Winter’s procedure. Developed corpora fibrosis
Dodds et al. [6]	58 years old, 0.4 mg per day for LUTS. Concomitant hydrochlorothiazide for hypertension. Priapism after fourth dose	Duration of erection: 7 h. Treated successfully with cavernosal aspiration and irrigation with a phenylephrine solution. Tamsulosin quitted. No new episodes until 6 months of follow-up
Kilink et al. [8]	59-years old, partial priapism started 2 h after the ingestion of the second dose of tamsulosin (0.4 mg), for LUTS. No history of trauma, sexual activity, bicycle riding, alcohol or drug use and no previous priapism.	Duration of erection: 2 days. Complete detumescence after irrigation of the corpus cavernosum with saline and proximal corpus cavernosal-spongiosum shunt. Patient was potent after 3 months
Marconi et al. [9]	45 years old. Priapism after 2 days, dosage of Ketorolac 10 mg three times a day and tamsulosin 0.4 mg once a day for renal colic episode. No concomitant diseases or use of medications.	Duration of erection: 6 h. Detumescence was achieved with five boluses injection to the corpora cavernosa of 200 microgram phenylephrine in 2 mL solution. No new priapism episode and no problems with erections and sexual intercourse

This case report has been reported according to the SCARE criteria [11].

## 2 Case presentation [11]

A 57-year-old man, presented in an emergency at our institution on December 11th, 2017 with a persistent painful erection 72 h in duration after failed conservative treatment. He gave history of recurrent painful penile erection every time he took a dose of tamsulosin (0.4 mg), each morning lasting for about half an hour. The complaints developed after the ingestion of medication, as prescribed for the treatment of lower urinary tract symptoms related to benign prostatic obstruction 2 years before. During the interview, the patient denied history of genital trauma, sexual activity, ingestion of alcohol or any illegal substances. Other causes of priapism were unlikely, as medical evaluation did not show any of associated medical conditions and previous episodes of painful erections. The other concurrent medication was ace inhibitor, beta blocker, and statin for hypertension and dyslipidemia treatment.

Physical examination revealed an erect penis with hard area located at along the proximal part of the penile shaft (Graphic 1). There was neither ﬂuctuation nor induration on palpation. There were no signs of erection in the distal part of the affected side, or the glans, and these areas were soft on palpation. Laboratory analysis revealed no signs of infection, hematological disease or malignancy. The urine analysis was unremarkable. The patient then scheduled for surgery.

**Graphic 1:**
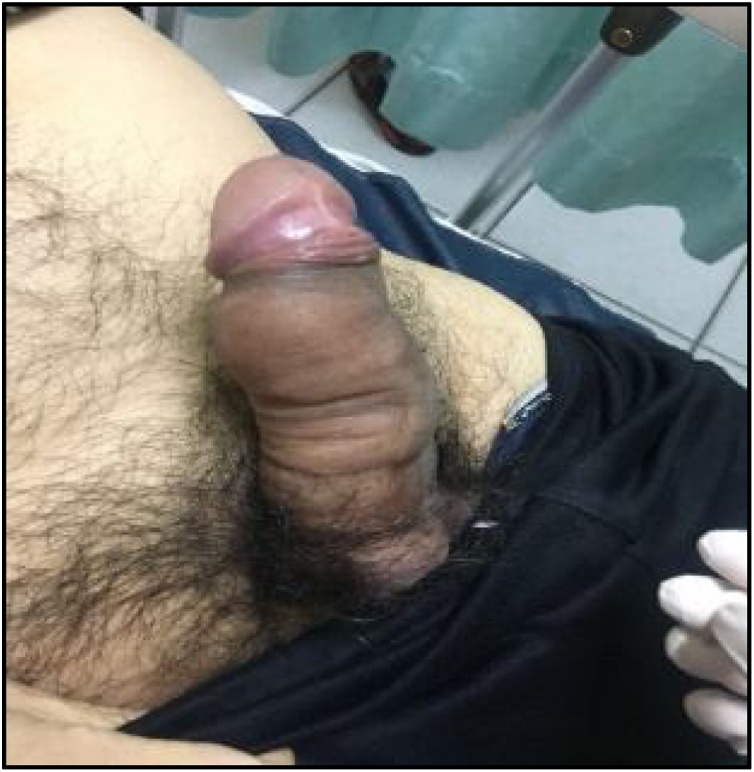
Pre operative.

Patient had a spinal anesthesia and positioned supine. First, we performed aspiration and irrigation of the corpus cavernosum from the lateral side with saline-diluted adrenaline solution ne (dosage of 2 mL of 1/100,000) up to five times over a 20-minute period (Graphic 2), but it was unsuccessful. Distal transglandular cavernosal aspiration and irrigation was then performed (Graphic 3). We used two 16 G needles. Our interference led to complete detumescence after 30 min.

**Graphic 2 and 3:**
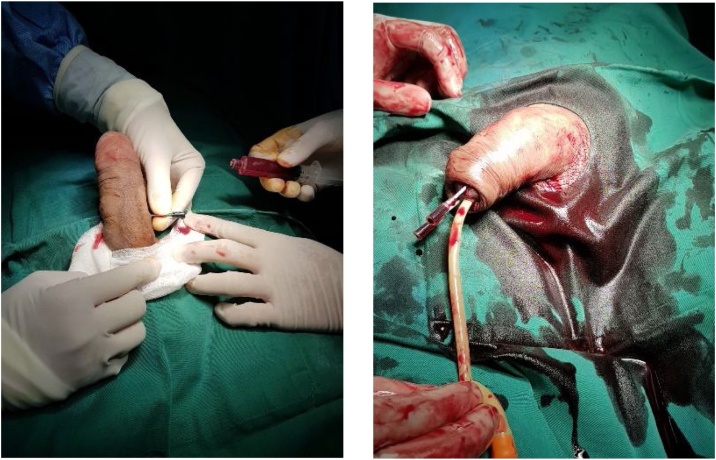
Intra Operative.

During the surgery, the dark viscous blood was aspirated from the corpora, and pH value of the aspirated blood was determined as 6.87; pCO2 was 73.0 mmHg and pO2 1.4 mmHg, HCO3 13 mmol/L, BE-ecf -23%, and O2 sat 5% indicating hypoxia. No serious problems occurred during the post-operative period (Graphic 4), the patient was discharge 5 days after the operation. The drug was discontinued and at follow-up over a 2-month period, the patient’s course was uneventful.

**Graphic 4:**
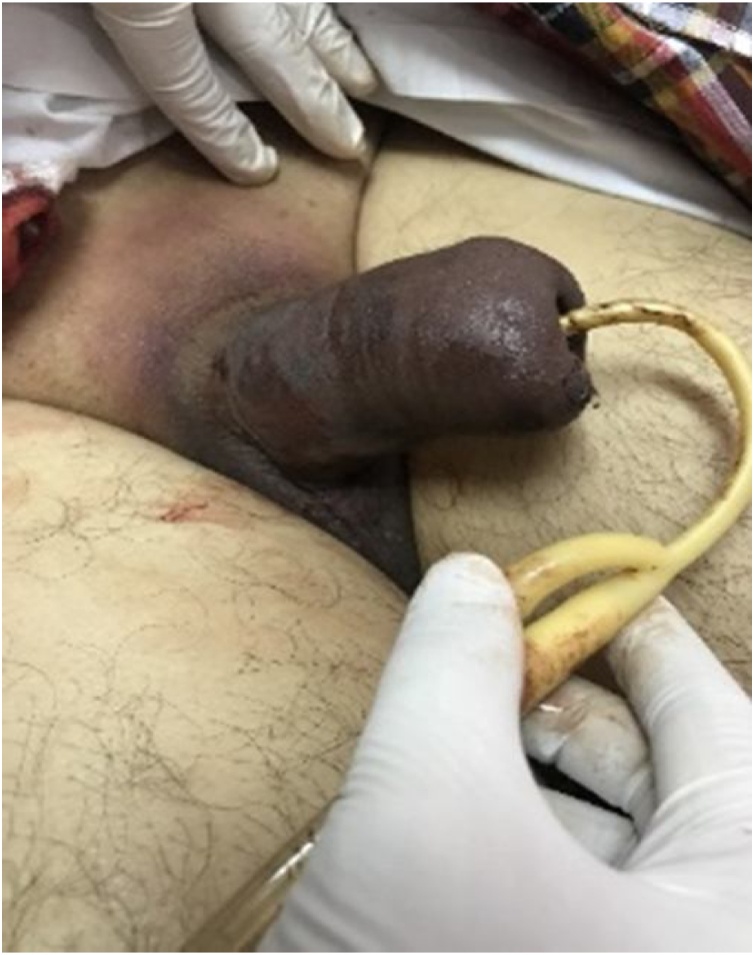
Post operative.

## 3 Discussion

Adrenergic a-blockers were first developed to control blood pressure, but due to their effect on prostatic stroma and capsule relaxation, they were then used for urological practice and became the primary treatment of the lower urinary tract symptoms (LUTS) caused by the prostate obstruction [4,5,7]. Alpha-adrenergic blockers inhibit the sympathetic effects necessary for the detumescence of the penis and thus lead to priapism [1,5]. Most of alpha adrenergic blockers such as Prazosin, Doxazosin, Terazosin, and Tamsulosin have been reported to cause induced priapism [1,5]. While cases of priapism has been observed in patients using tamsulosin, but tamsulosin is the uroselective drug and the mainstay used today in urological practice because it has the least side effect with very rare reports of priapism [9]. Reports was found in the context of older patients that applied in higher doses for the treatment of hypertension [7].

Tamsulosin is a selective subtype of alpha blockers works effectively on the prostate, but it appears that it might also has an effect on the corporal smooth muscle [1,8]. Tamsulosin is the only alpha adrenergic blocker with a placebo-controlled study showing that it improves the sexual function suggested that priapism might develop as a result of the secondary effect [1]. Hence, their argument was that the observed priapism could be interpreted as the extreme end of a spectrum of manifestations of an otherwise desired phenomenon and that concomitant drugs that inhibit its metabolism or intake of high doses of tamsulosin may result in priapism [5].

One case described the association of tamsulosin to a drug (Boceprevir-CYP3A4) that inhibits its degradation triggered a priapism episode [10]. When analyzing our case, we were not able to find any other etiology of priapism in our patient. He was healthy with no hematological or neurologic diseases, while medications he had consumed were antihypertensive therapy and tamsulosin. There are no reports in the literature of the association between antihypertension with the development of priapism.

## 4 Conclusion

Tamsulosin is an uroselective drugs commonly used for the treatment of LUTS associated with BPH and medical expulsion therapy of distal ureterolithiasis. The incident of priapism related to this class of drugs is rare. However, with the increasing use of this drug, it would not be surprising to see more cases of tamsulosin-induced-priapism in the future. Upon prescription of tamsulosin, physicians should be aware of the possible complication. Patients should be informed about the possibility of this complication, and also educated that upon initial observations of painful involuntary erections, regarding the golden period, to stop the drug and seek medical attention as soon as possible.

With caution against the use of tamsulosin in hypertension treated patient, the possibility of the adverse effect can be more noticed and also encourage practitioners to look for other alternatives and factors that may lead to increased risk of alpha-blocker-induced priapism in the future and develop better treatment strategies and a recommendation of avoiding this drugs when on tamsulosin.

## Declaration of Competing Interest

We don’t have any conflicts of interest.

## Funding

We fund the research all by ourselves.

## Ethical approval

We hereby state that we have the approval from our Hospital Ethical Committee and the patient himself.

## Consent

Written informed consent was obtained from the patient for publication of this case report and accompanying images.

## Author contribution

Study Conception and Design: Prihadi, Kusumajaya.

Acquisition of Data: Prihadi, Kusumajaya.

Analysis and Interpretation of Data: Prihadi, Kusumajaya.

Drafting of Manuscript: Prihadi, Kusumajaya.

Critical Revision: Prihadi, Kusumajaya.

## Registration of research studies

Our Case Report did not invole any human trials or studies.

## Guarantor

Johannes Cansius Prihadi, MD, PhD.

## Provenance and peer review

Not commissioned, externally peer-reviewed.
